# High-Avidity Anti-Filovirus IgG Elicited Using Protein Subunit Vaccines Does Not Correlate with Protection

**DOI:** 10.3390/immuno3040022

**Published:** 2023-10-24

**Authors:** Caitlin A. Williams, Teri Ann S. Wong, Michael M. Lieberman, Jake Yalley-Ogunro, Mehtap Cabus, Sara Nezami, Fabian Paz, Hanne Andersen, Thomas W. Geisbert, Axel T. Lehrer

**Affiliations:** 1 Department of Tropical Medicine, Medical Microbiology and Pharmacology, John A Burns School of Medicine, University of Hawaii at Manoa, Honolulu, HI 96813, USA;; 2 BIOQUAL, Inc., Rockville, MD 20850, USA;; 3 Galveston National Laboratory, Department of Microbiology and Immunology, University of Texas Medical Branch, Galveston, TX 77555, USA;

**Keywords:** vaccines, filovirus, avidity, nonhuman primates

## Abstract

Zaire ebolavirus (EBOV) poses a significant threat to public health due to its high case fatality rate and epidemic potential. This is further complicated by the lack of precise immune correlates of protection and difficulties in conducting in vivo animal studies due to species specificity of Ebola virus disease (EVD) and classification as a biosafety level 4 pathogen. Related ebolaviruses have also contributed to the public health threat; Uganda recently experienced an outbreak of Sudan ebolavirus, which also had a high case fatality rate. Vaccination targeting EBOV has demonstrated significant efficacy; however, the protective cellular and humoral responses at play are still poorly understood. Vaccination for vulnerable populations such as pregnant women, young children, and immunocompromised individuals is still limited. Understanding vaccine correlates of protection (vCOP) is key to developing alternative vaccination strategies for these groups. Components of immunity such as neutralizing antibody and cell-mediated immunity are likely responsible for protective responses; however, existing research fails to fully define their roles in protection. Here we investigated vaccine-elicited antibody avidity as a potential correlate of protection and to further characterize the contribution of antibody avidity in protective and nonprotective vaccine responses.

## 1. Introduction

Currently, the only vaccine options to prevent Ebola virus disease (EVD) are viral vectored-vaccines Ervebo and Zabdeno in combination with Mvabea [1]. Ervebo utilizes vesicular stomatitis virus, expressing the EBOV GP as the surface protein to induce protective efficacy in humans in a single dose [2]. Zabdeno utilizes a heterologous prime boost in which the primary dose consists of recombinant adenovirus expressing EBOV GP [3]. The boost dose consists of recombinant MVA encoding for the GP of SUDV, MARV, EBOV, and the nucleoprotein of Tai Forest virus. The duration of protective efficacy for these vaccines is currently unknown, as there are no known vaccine correlates of protection. Vaccine correlates of protection (vCOP) are defined as measurable immunological responses that correlate with statistical significance to a second measurable aspect of protection such as decreased viremia, or survival day after infection [4]. Determining the vCOP for Ebola is dependent on a thorough understanding of immune correlates derived from vaccines, correlates of survival from Ebola survivors, as well as correlates of protection in preclinical vaccine trials [4]. Historically, the nature of filovirus infection creates a particular challenge in assessing survivors since infection yields a high case fatality rate, coupled with outbreaks occurring in rural areas of Central Africa, making access to healthcare a significant barrier for patients and researchers. The EVD outbreak of West Africa in 2013–2016 occurred in an urban region, which enabled patients to access healthcare more readily; however, the population density facilitated disease transmission, causing a largescale and prolonged outbreak. As a result of this outbreak, researchers learned that survivors demonstrated less pronounced inflammation with the activation of CD8+ T cells, upregulation of type 1 IFN, a lower concentration of circulating monocytes, and lower viral load, whereas patients who succumbed to EVD experienced a CD4+, CD8+ T cell response with high expression of CTLA-4 and higher viral load. The IgG response in survivors demonstrated that Ebola survivors develop a class-switched humoral response comprising IgM, IgA, and IgG with predominant specificity for the GP and VP40. The humoral response is limited in early infection but becomes more robust with time, suggesting affinity maturation in those who survive beyond week 1 of symptom onset [5].

To more fully understand the characteristics of a protective immune response that can serve as vCOP in humans, our group conducted several preclinical studies in nonhuman primates [6]. Utilizing recombinant protein subunit vaccines adjuvanted with a novel oil in water emulsion adjuvant, we established a vaccine platform capable of fully protecting nonhuman primates upon viral challenge with a lethal dose of the Kikwit strain of EBOV. To determine vCOP and further characterize the humoral response elicited by our vaccines, we compared the avidity of IgG developed by animals that survived viral challenge and those that did not. The cohort investigated here comprises a fully protective schedule, consisting of three doses of vaccine followed by immediate challenge, or two doses of monovalent vaccine with a slightly later challenge (seven versus four weeks after last dose), and two regimens with lower efficacy corresponding to a two-dose multivalent formulation with slightly delayed challenge and a three-dose regimen with a 1-year delay prior to viral challenge (Table 1). Considering the difference in binding of licensed immunotherapies, REGN-EB3, a cocktail of three human monoclonal antibodies [7], and mAb114, a single human monoclonal antibody [8,9], compared to an immunotherapy that failed to reach licensure, ZMAPP [10,11], we sought to investigate avidity and binding under low pH and chaotropic conditions of protective, partially protective, and nonprotective humoral responses. Here, we demonstrate that while protein subunit vaccines elicit a high avidity antibody, differential binding under chaotropic conditions was not a predictor of survival.

## 2. Materials and Methods

### 2.1. Vaccines and Nonhuman Primate Immunizations

Serum collected from previously completed studies were utilized for this study [6]. To summarize these studies, *Zaire ebolavirus* glycoprotein (EBOV GP), *Sudan ebolavirus* glycoprotein (SUDV GP), *and Marburg marburgvirus* glycoprotein (MARV GP) were produced recombinantly in Drosophila S2 cells as previously described [12,13]. Twenty-five micrograms of EBOV GP (monovalent formulation) or 25 μg of each GP (trivalent formulation) were mixed with CoVaccineHT^™^ (Protherics Medicines Development Ltd., London, UK) and administered in both deltoids (split dose) of cynomolgus macaques. Monkeys were immunized with two or three doses of vaccine, each dose 3 weeks apart. Monkeys were either challenged at week 10 or week 62 depending on the study. Serum samples were collected for immunogenicity analysis two weeks after each dose. Monkeys were challenged with 1000 PFU of low-passage EBOV, Kikwit isolate. Monkeys were humanely euthanized upon exhibiting signs and symptoms of EVD, specifically a decrease in appetite, fever; increase in AST, ALT, CRE, BUN; development of petechiae, viremia; and a drop in platelet counts.

### 2.2. Quantification of Antigen-Specific IgG

A multiplex bead array immunoassay was used to determine total antigen-specific IgG. Antigen-specific immunoglobulin concentrations in monkey sera were measured as previously described [14]. Briefly, this assay comprises magnetic beads covalently linked to antigen, which include glycoproteins of EBOV, SUDV, and MARV, and bovine serum albumin (BSA) used as a negative control. MagPix beads were combined in MIA buffer (1x PBS + 1% BSA + 0.02% Tween 20) to make a master mix suspension at a dilution of 1/200, and 50 μL of this microsphere suspension was added to each well of a black-walled, clear bottom, 96-well plate. Ten-fold serum dilutions were performed to reach a final dilution of 1/10,000. Each dilution of anti-EBOV GP IgG or serum was added to wells containing microspheres and incubated for 3 h shaking at 650 rpm in a 37 °C incubator. Plates were triple-washed with MIA buffer using a magnetic plate separator (Millipore Corp., Billerica, MA, USA). Samples were probed with 50 μL of red phycoerythrin (R-PE) conjugated F(ab’)2 fragment goat anti-human IgG (Jackson ImmunoResearch, Inc., West Grove, PA, USA) at 1/250 dilution and incubated for one hour at 37 °C with shaking at 650 rpm. Plates were then washed three times as described above and 120 μL of MagPix drive fluid (Luminex Corp., Austin, TX, USA) was subsequently added to each well. Plates were analyzed using the MAGPIX Instrument (Luminex Corop., Austin, TX, USA). Data acquisition for detection of the median fluorescence intensity (MFI) was set to 50 beads per bead region. Antigen-coupled beads were recognized and quantified based on bead fluorescent region and signal intensity, respectively. Data were graphed using Prism GraphPad v9 (Boston, MA, USA) and Microsoft Excel, Microsoft Corporation (Redman, WA, USA) (2011).

### 2.3. Avidity Assay

To determine antibody avidity, a multiplexed assay was conducted as described above. After 3 h, incubation plates were washed as above. Increasing molarities of the anionic chaotrope NH4SCN (0 M, 0.5 M, 1 M, 2 M, 4 M, or 6 M) were added to the plate. Either NH4SCN or low pH buffer was added to each well; for pH-based assays, PBS was buffered to a pH of either 7.4, 6, 5.5, or 4.5. Plates were incubated for 30 min at 37 °C, shaken at 650 rpm. Subsequently, plates were washed three times with MIA buffer. Samples were probed with R-PE-labeled goat anti-human IgG antibody as described above. A relative avidity index was calculated by taking the ratio of the MFI of the 2 M NH4SCN or each pH condition-treated sample to the untreated sample and multiplying by 100.

(1) (MFIwithtreatment/MFIwithouttreatment)×100%

### 2.4. Statistical Analysis

Spearman correlations were conducted between relative avidity index, total IgG, and survival day. The Mann–Whitney test was used to calculate statistical differences between survivors and non-survivors. All statistical analyses were conducted using Prism GraphPad v9 (Boston, MA, USA).

## 3. Results

### 3.1. Avidity Maturation Peaks with Three Doses of Vaccine

**Figure 1.**
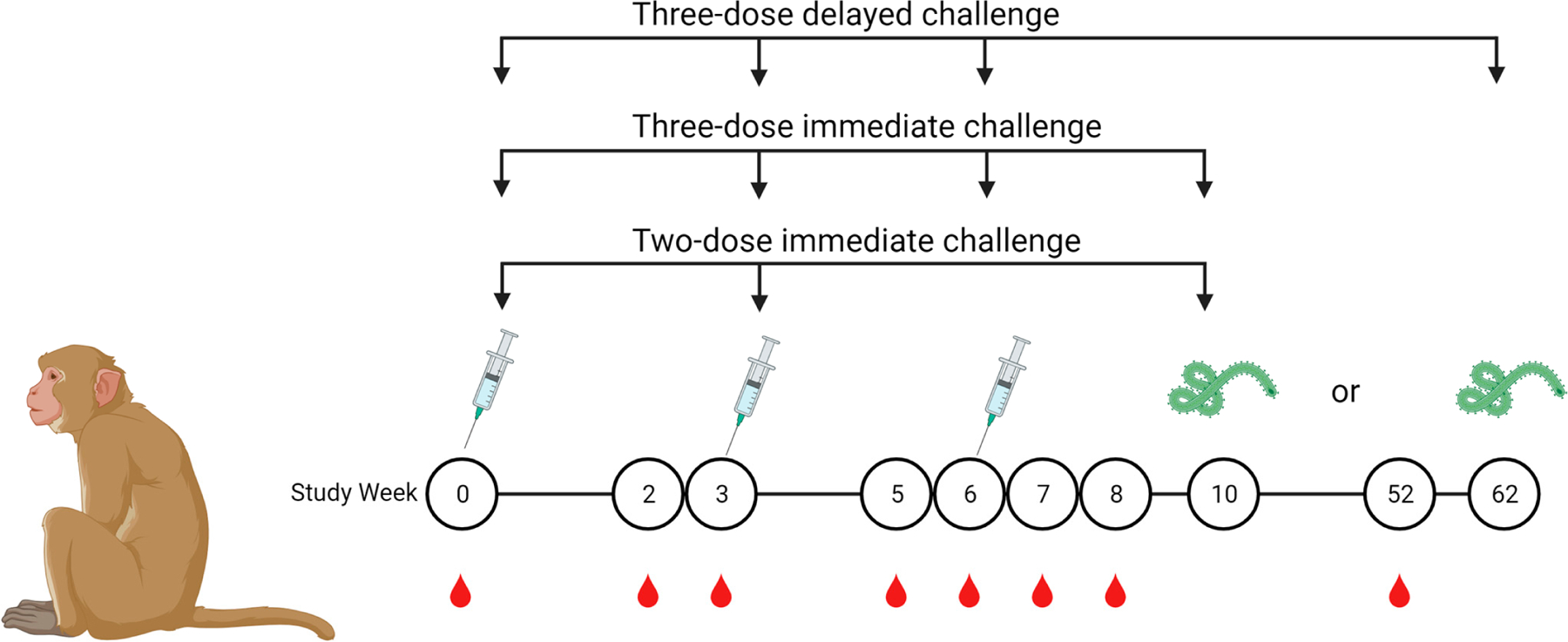
Immunization, blood draw, and challenge schedule. Cynomolgus macaques were immunized on either a two-dose or three-dose schedule and challenged with a lethal dose of Zaire ebolavirus, Kikwit strain, at week 10 or 62. Serum for MIA was collected as indicated by red droplets.

**Figure 2.**
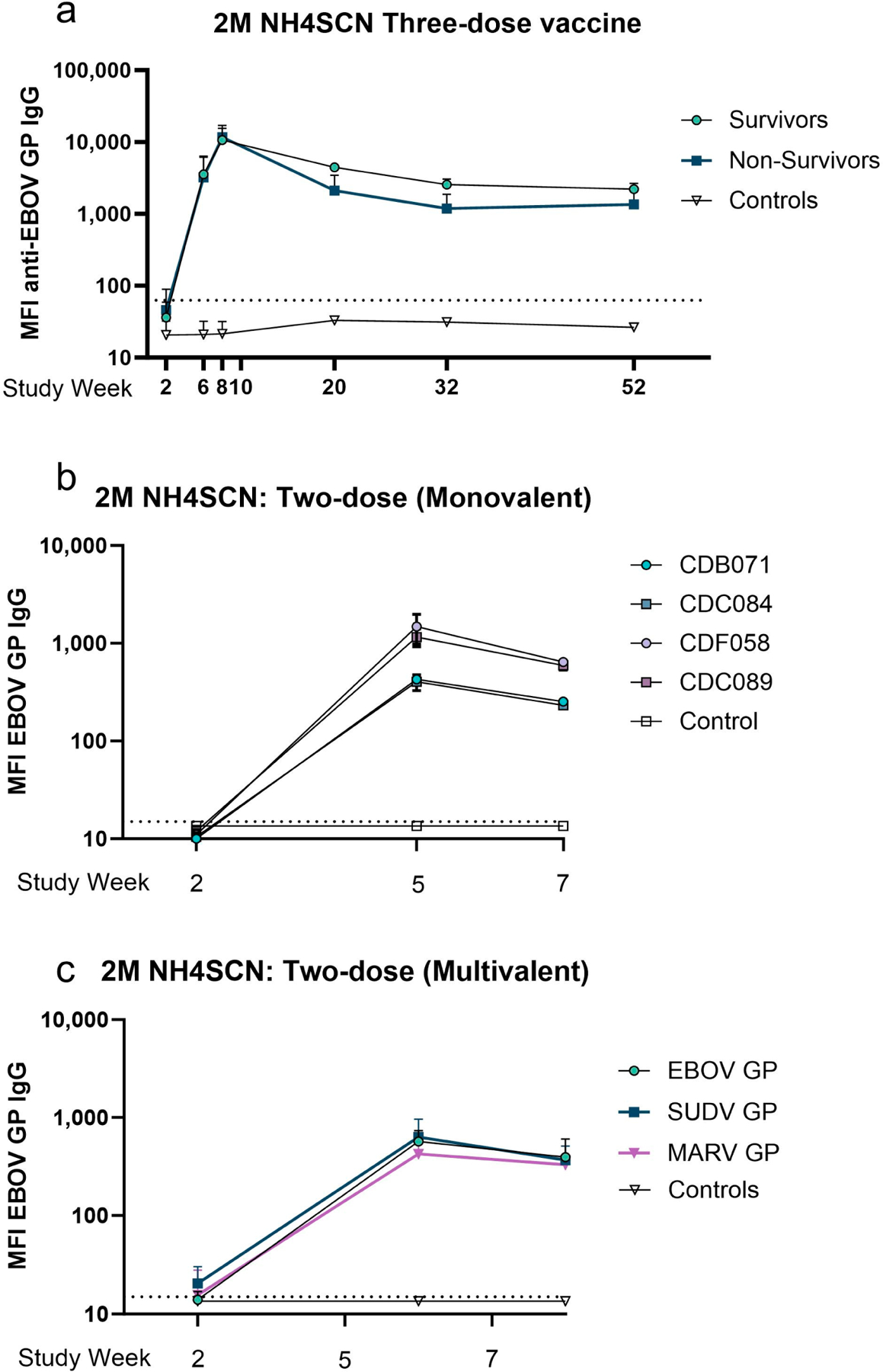
Effect of treatment with 2 M NH4SCN on antibody binding to antigen. (**a**–**c**) Serum samples collected were diluted 1:10,000 and treated with a 2 M NH4SCN wash during MIA assay. A negative cutoff was calculated as the average of the control plus three standard deviations, indicated by a grey dotted line. Datapoints represent the mean of replicate assays with error bars of the standard deviation. (**a**) MFI of EBOV GP-specific IgG after treatment. In a three-dose cohort, serum samples were collected two weeks after each dose and intermittently for one year. Eight animals were challenged at week 10 and eight were challenged at week 62, including two controls (nonvaccinated) at each timepoint. Datapoints represent the median MFI of animals grouped by challenge outcomes or as controls (survivors, *n* = 2; non-survivors, *n* = 4; control, *n* = 2). (**b**) MFI of antigen-specific IgG after treatment in a two-dose monovalent cohort. Serum samples were collected two weeks after each dose of vaccine. Datapoints represent the average MFI of duplicate assays. (**c**) MFI of anti-EBOV, SUDV, or MARV GP IgG after treatment in a two-dose multivalent cohort. Serum samples were collected two weeks after each dose. All samples were run in duplicate.

We selected a cohort comprising protected, partially protected, and nonprotected monkeys based on the viral challenge outcomes of our previous efficacy studies [6] to conduct an analysis of antibody avidity maturation and proportional analysis of avidity one year postimmunization. This study is comprised of monkeys immunized with a vaccine on either a two-dose or a three-dose vaccine schedule with three-week intervals between doses. Monkeys administered a monovalent three-dose immunization were challenged either on week 10 or week 62. The study schedule is detailed in Table 1 and Figure 1. Serum samples were collected at multiple timepoints during the vaccine schedule; samples collected at two weeks after each dose were analyzed using a multiplexed immunoassay with a chaotrope wash. Samples were treated with an anionic chaotrope, 2 M NH4SCN. Animals that received three doses of monovalent vaccine had greater fluorescence intensity remaining after the chaotrope wash than monkeys that received only two doses of vaccine, indicating potential for stronger avidity. In the three-dose cohort, six animals were challenged at study week 10, and six were challenged at study week 62 (Figure 1). Antibody detected after a wash with 2 M NH4SCN continued to increase after a third dose of vaccine, with slight waning over the one-year rest period. (Figure 2a). Animals that received two doses of vaccine demonstrated a similar trend (Figure 2b). Antibody levels against all three antigens in the vaccine were comparable to one another, indicating similar avidity maturation of all three antigen-specific antibodies; however, these levels declined slightly before viral challenge (Figure 2c).

### 3.2. Avidity of GP-Specific Antibodies from Cynomolgus Macaques Increases with Increasing Number of Vaccine Doses

**Figure 3.**
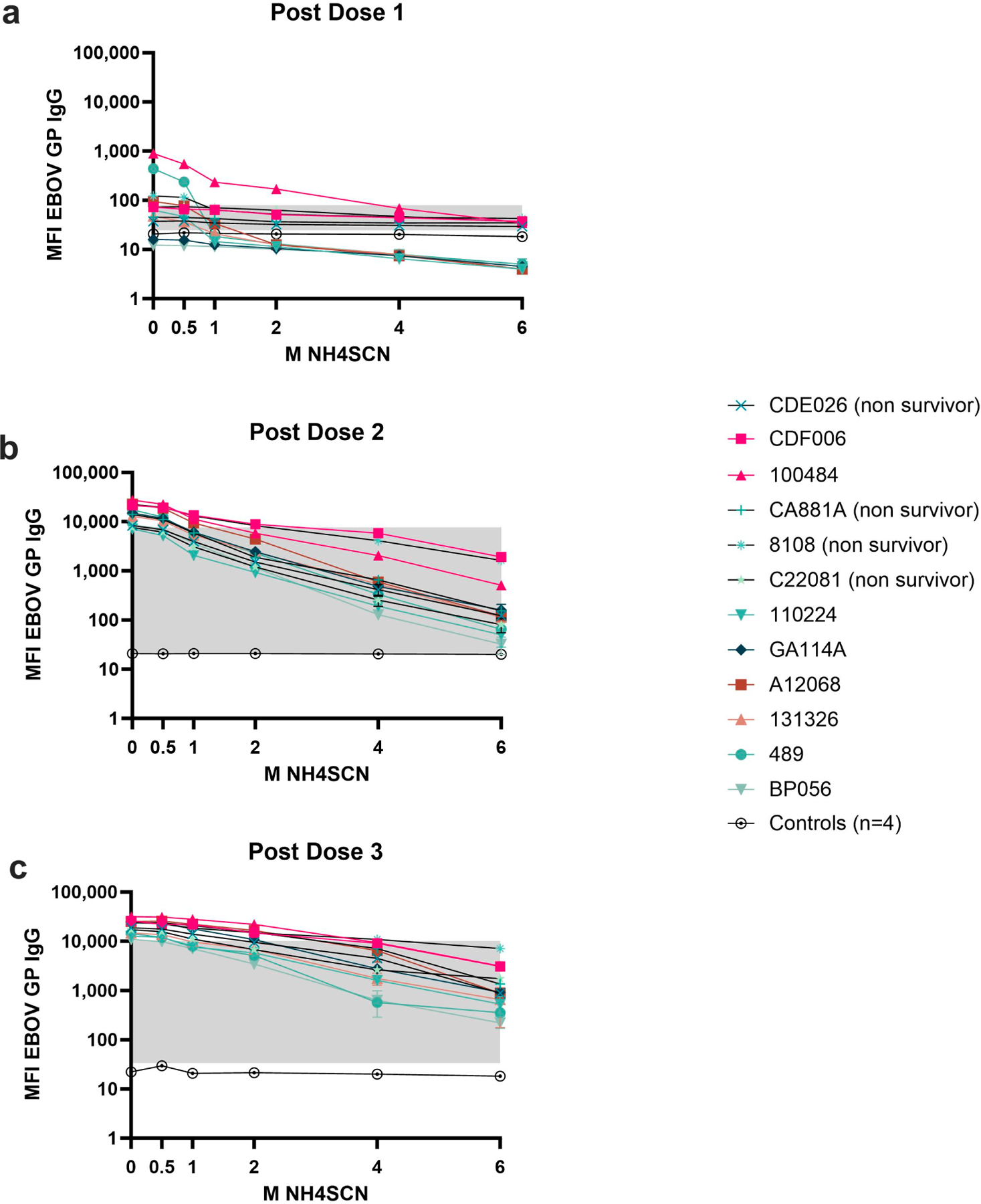
Antibody avidity after each dose of vaccine. Serum samples were collected two weeks after each dose of vaccine. Animals were given a total of three doses of monovalent EBOV vaccine. Serum samples were treated with either 0.5 M, 1 M, 2 M, 4 M, or 6 M NH4SCN. The grey box indicates the space occupying <50% of untreated binding and the negative cutoff. The negative cutoff was calculated as the average of the MFI of the control animals plus three standard deviations. (**a**) Dose 1 response; (**b**) dose 2 response; (**c**) dose 3 response.

Serum samples collected after each vaccine dose were subjected to MIA avidity assays using increasing molarities of NH4SCN up to 6 M (Figure 3). Antibody avidity after the first dose of vaccine was weak; monkeys developed low antibody levels to EBOV GP, and NH4SCN concentrations < 1 M could remove 50% of antibody compared to an untreated sample (Figure 3a). 1 M NH4SCN could remove all antibody and levels began to approach the negative cutoff for 11/12 animals. Animal CA881A developed strong binding antibody after the first dose and did not survive the viral challenge (Figure 3a). A second dose of vaccine elicited avidity maturation, yielding stronger binding antibody; 2 M NH4SCN removed about 50% of antibody in the assay at this timepoint and overall antibody levels remained above the negative cutoff (Figure 3b). After a third dose of vaccine, both the immediate and delayed challenge groups developed high-avidity responses (Figure 3c). Animals had a less varied response and retained antibody levels well above the negative cutoff even with 6 M NH4SCN treatment.

### 3.3. Antibody Avidity Remains High during a One-Year Rest Period Prior to Viral Challenge

**Figure 4.**
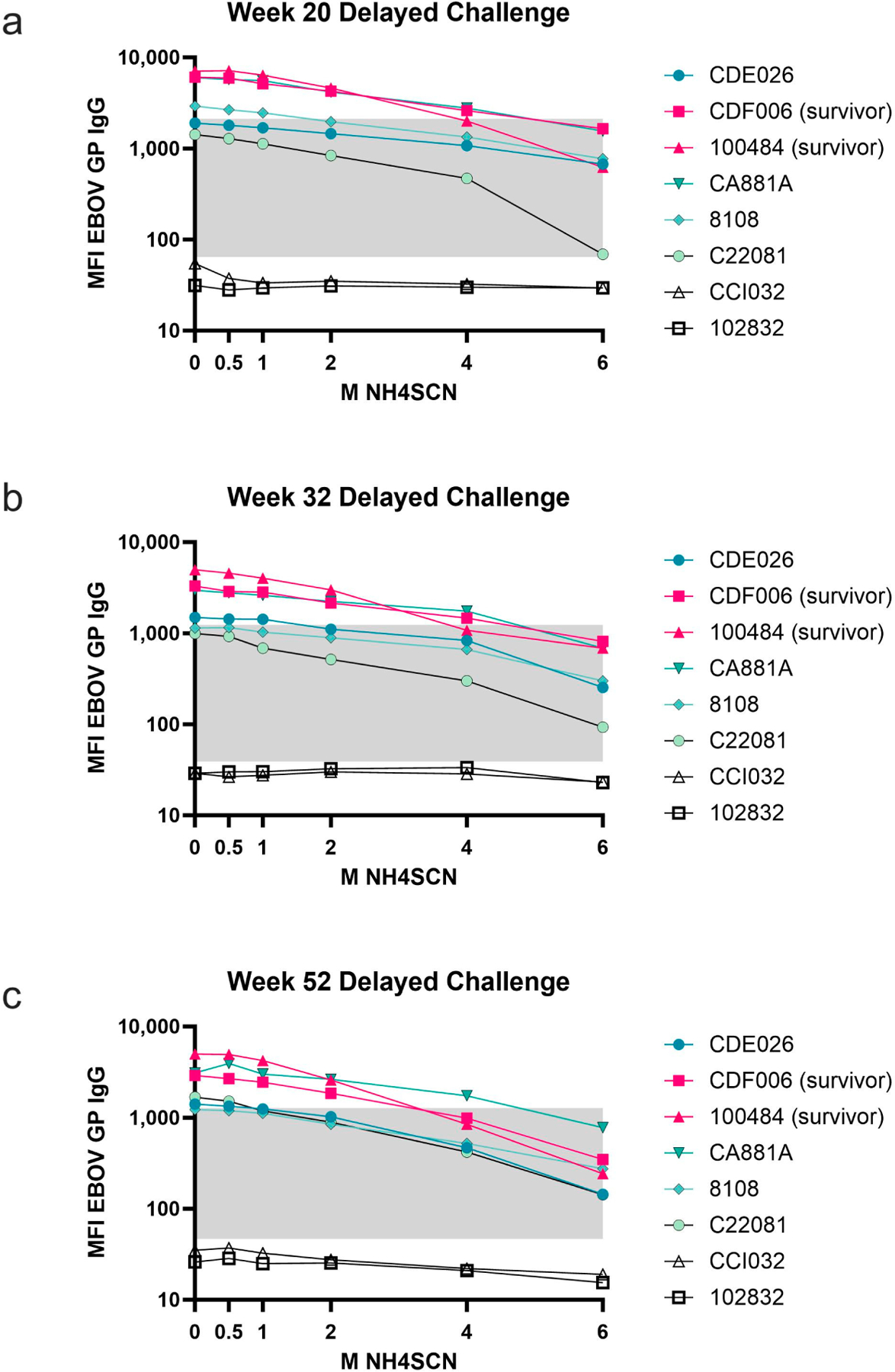
Avidity during 1-year rest period. Serum samples taken at the indicated timepoints postimmunization (weeks 20, 32, and 52) were subject to MIA with either 0.5 M, 1 M, 2 M, 4 M, or 6 M NH4SCN. Survivors are in pink, non-survivors are in teal. The grey box indicates the space occupying <50% of untreated binding and the negative cutoff. The negative cutoff was calculated as the average of the MFI of the control animals plus three standard deviations. (**a**) Serum samples collected at study week 20. (**b**) Serum samples collected at study week 32. (**c**) Serum samples collected at study week 52 (10 weeks prior to viral challenge).

During the one-year rest period, antibody avidity remained strong, but with some variability among animals (Figure 4). There was an observed overall decrease in total antigen-specific IgG during the one-year rest period; however, treatment with as much as 6 M NH4SCN did not remove a significant amount of antibody from any of the animals except C22081 (Figure 4a); this animal did not survive the viral challenge. Animals that survived the viral challenge had slightly higher avidity than non-survivors. However, one animal that did not survive the viral challenge developed an antibody that remained bound at a higher molarity of NH4SCN than the protected animals (Figure 4c). During the one-year rest period, antibody levels decreased and therefore the bound antibody after treatment with 2 M NH4SCN decreased as well (Figure 4a–c).

### 3.4. A Two-Dose Recombinant EBOV GP Vaccine Regimen Generates Moderate Antigen-Specific Avidity

**Figure 5.**
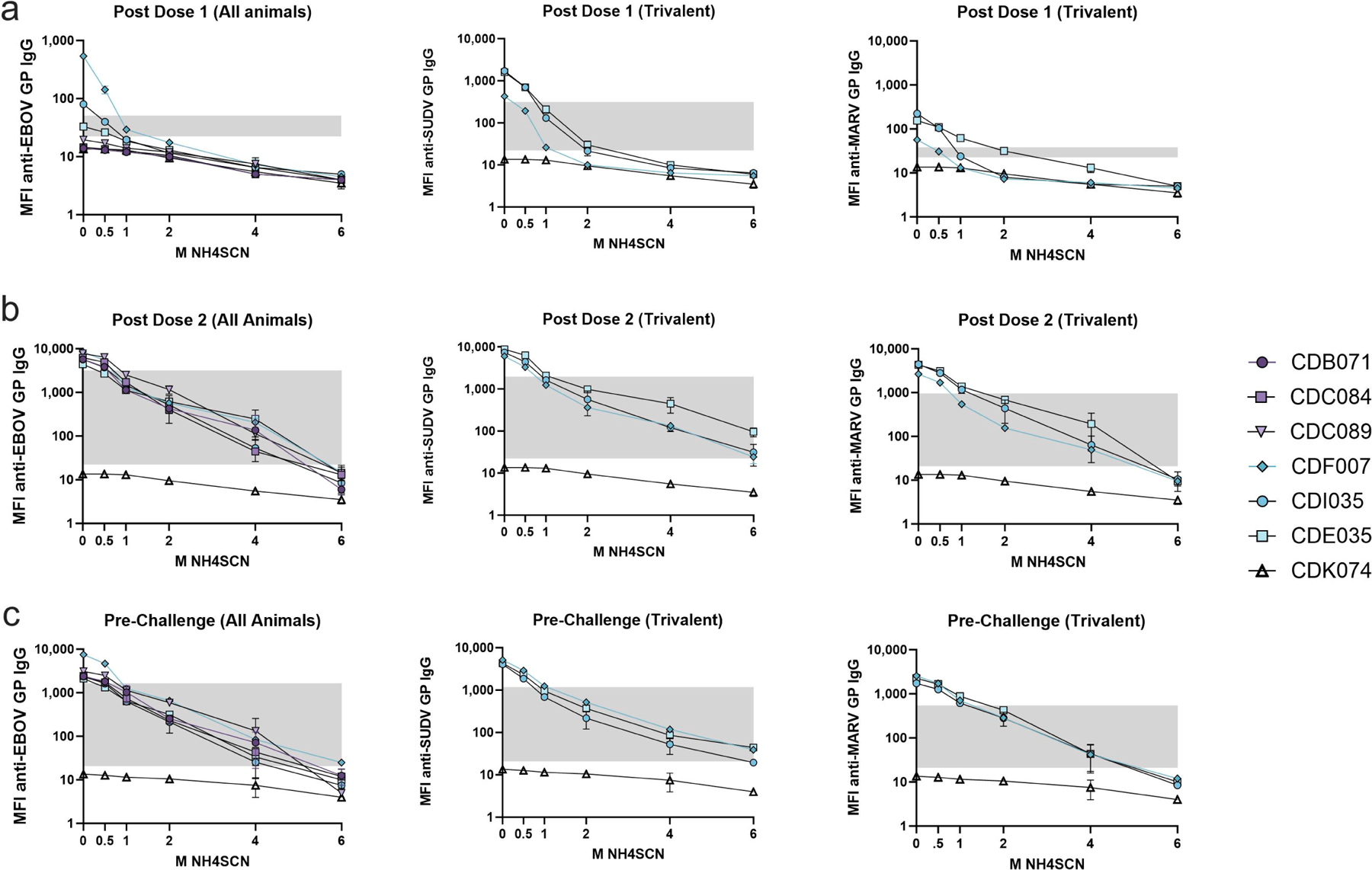
Avidity during the two-dose vaccine schedule. Serum was collected two weeks after each dose and prior to challenge (week 7). Serum samples were diluted 1:10,000 and subjected to MIA with a chaotrope wash with either 0.5 M, 1 M, 2 M, 4 M, or 6 M NH4SCN. The grey box indicates the space occupying < 50% of untreated binding and the negative cutoff. The negative cutoff was calculated as the average of the MFI of the control animals plus three standard deviations. (**a**) Serum samples collected from NHP two weeks after a dose of either monovalent or trivalent vaccine. (**b**) Serum samples collected from NHP two weeks after the second dose of either a monovalent or a trivalent vaccine. (**c**) Serum samples collected 1 week after a second dose. The first graph in each panel (anti-EBOV GP) contains the data from both the monovalent and trivalent vaccine groups. The second and third graphs in each panel (anti-SUDV GP and anti-MARV GP, respectively, contain data from only the trivalent vaccine group.

We next evaluated the antibody avidity of monkeys immunized with two doses of either monovalent or trivalent vaccine. Serum collected two weeks after each dose and three weeks prior to challenge from both cohorts was analyzed using MIA with treatment with either 0.5 M, 1 M, 2 M, 4 M, or 6 M NH4SCN (Figure 5). EBOV GP-specific antibody binding was measured for both cohorts. Both monovalent and trivalent recipients developed low-avidity antibody after one dose (Figure 5a). After a second dose of either monovalent or trivalent vaccine, overall binding strength to the EBOV GP increased (Figure 5b). At 4 M NH4SCN, all animals had detectable antibody levels remaining after treatment, although less than observed in the case of the three-vaccine dose group. Trivalent recipients underwent avidity maturation towards SUDV GP, and antibody levels remained above the negative cutoff with treatment of 6 M NH4SCN. The MARV GP response in the trivalent cohort had levels above the negative cutoff after treatment with 4 M NH4SCN; however, 6 M NH4SCN was able to remove nearly all the MARV GP IgG antibody after the second dose. (Figure 5b). Without a third dose of vaccine, antibody binding strength slightly decreased by week 10 (just prior to challenge); treatment with 4 M NH4SCN brought some antibody levels down to the negative cutoff. However, the overall patterns followed the same trend as the post-dose two response (Figure 5c).

### 3.5. Avidity of Cross-Reactive Antibody

**Figure 6.**
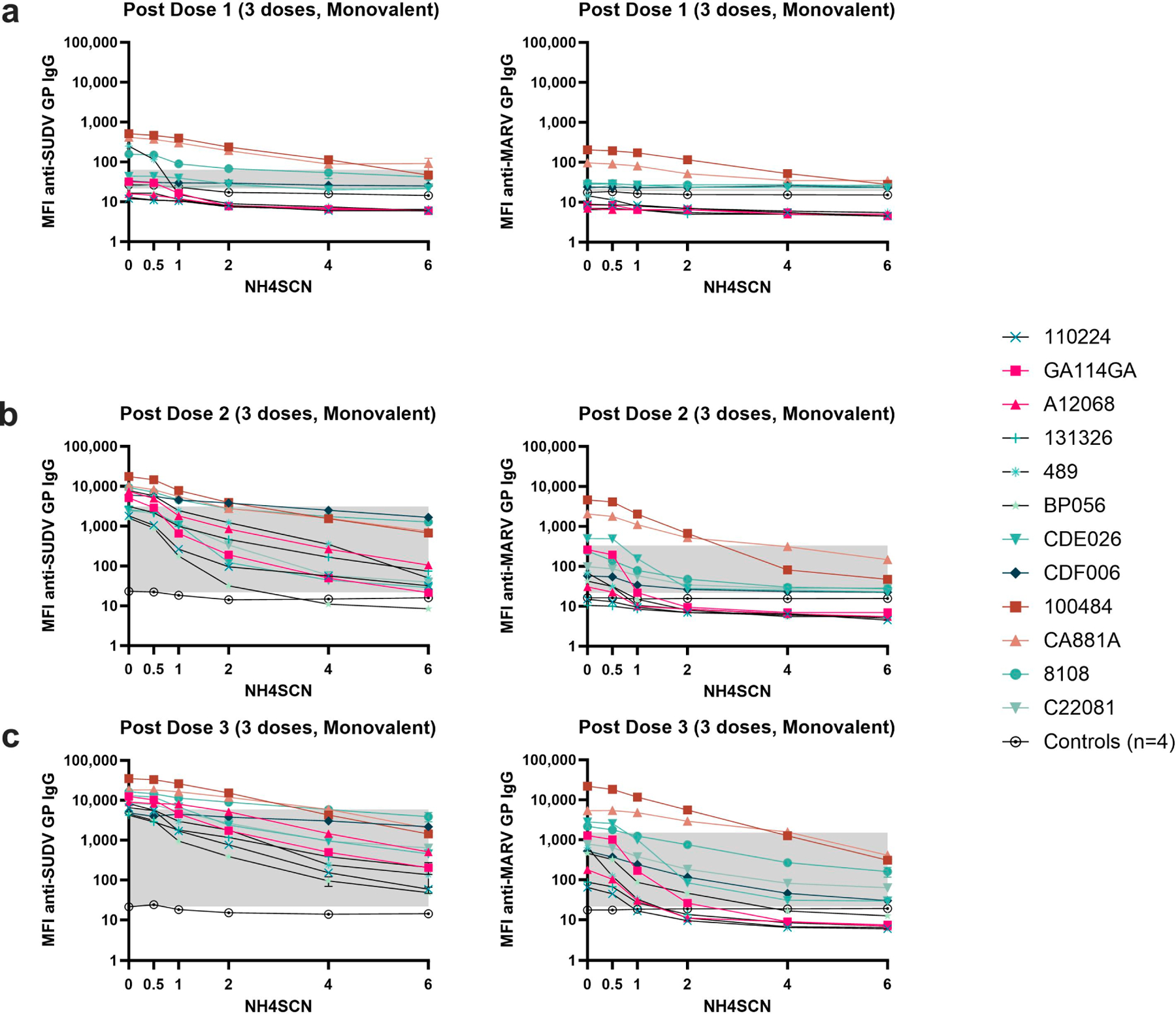
Avidity of cross-reactive IgG responses to a monovalent EBOV vaccine. Serum was collected two weeks after each dose of a monovalent EBOV GP vaccine. Serum samples were diluted 1:10,000 and subjected to MIA with a chaotrope wash with either 0.5 M, 1 M, 2 M, 4 M, or 6 M NH4SCN. The grey box indicates the space occupying < 50% of MFI without chaotrope treatment and the negative cutoff defining the lower end. The negative cutoff was calculated as the average of the MFI of the control animals plus three standard deviations. (**a**) Serum samples collected from NHPs two weeks after dose 1 of a monovalent EBOV vaccine. (**b**) Serum samples collected from NHPs two weeks after dose 2 of a monovalent EBOV vaccine. (**c**) Serum samples collected two weeks after dose 3 of a monovalent EBOV vaccine.

To assess the avidity of cross-reactive antibody, we measured antibody levels for SUDV GP and MARV GP after treatment with NH4SCN in the MIA. In the three-dose monovalent EBOV GP vaccine cohort, the SUDV GP cross-reactive antibody generated by the vaccine clearly increases in binding strength with each vaccine dose, indicating mutual avidity maturation (Figure 6a–c). However, the cross reactivity towards MARV GP does not exhibit the same degree of binding strength, generating lower antibody levels with lower avidity after three doses of vaccine compared to the anti-EBOV GP and anti-SUDV GP antibodies. Binding is highest in animals 100484, CA118A, and 8108 (Figure 6a–c).

**Figure 7.**
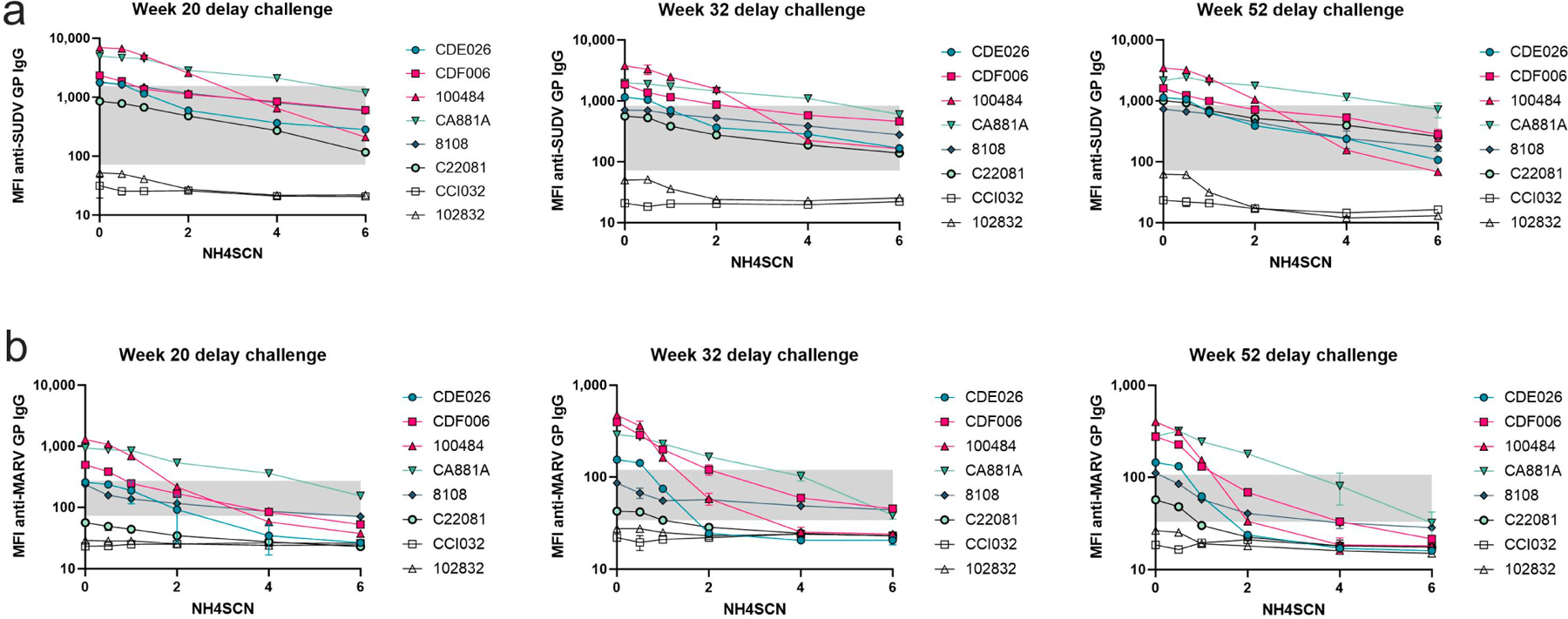
Cross-reactive avidity up to one year post immunization. Serum samples were collected at week 20, 32, and 52 during the one-year rest period prior to viral challenge and subjected to MIA with a chaotrope wash of either 0.5 M, 1 M, 2 M, 4 M, or 6 M NH4SCN. Antibody cross-reactive to SUDV GP and MARV GP was reported. (**a**) SUDV GP cross-reactive antibody; (**b**) MARV GP cross-reactive antibody. Animals CC1032 and 102832 were nonvaccinated controls.

During the one-year delay in the delayed challenge group, the cross-reactive antibody responses remained detectable with strong binding to the SUDV GP (Figure 7a). However, the MARV GP cross-reactive antibody response began to exhibit decreased binding strength in addition to overall decrease in antigen specific antibody levels after week 20 (Figure 7b).

### 3.6. Binding at Low pH

**Figure 8.**
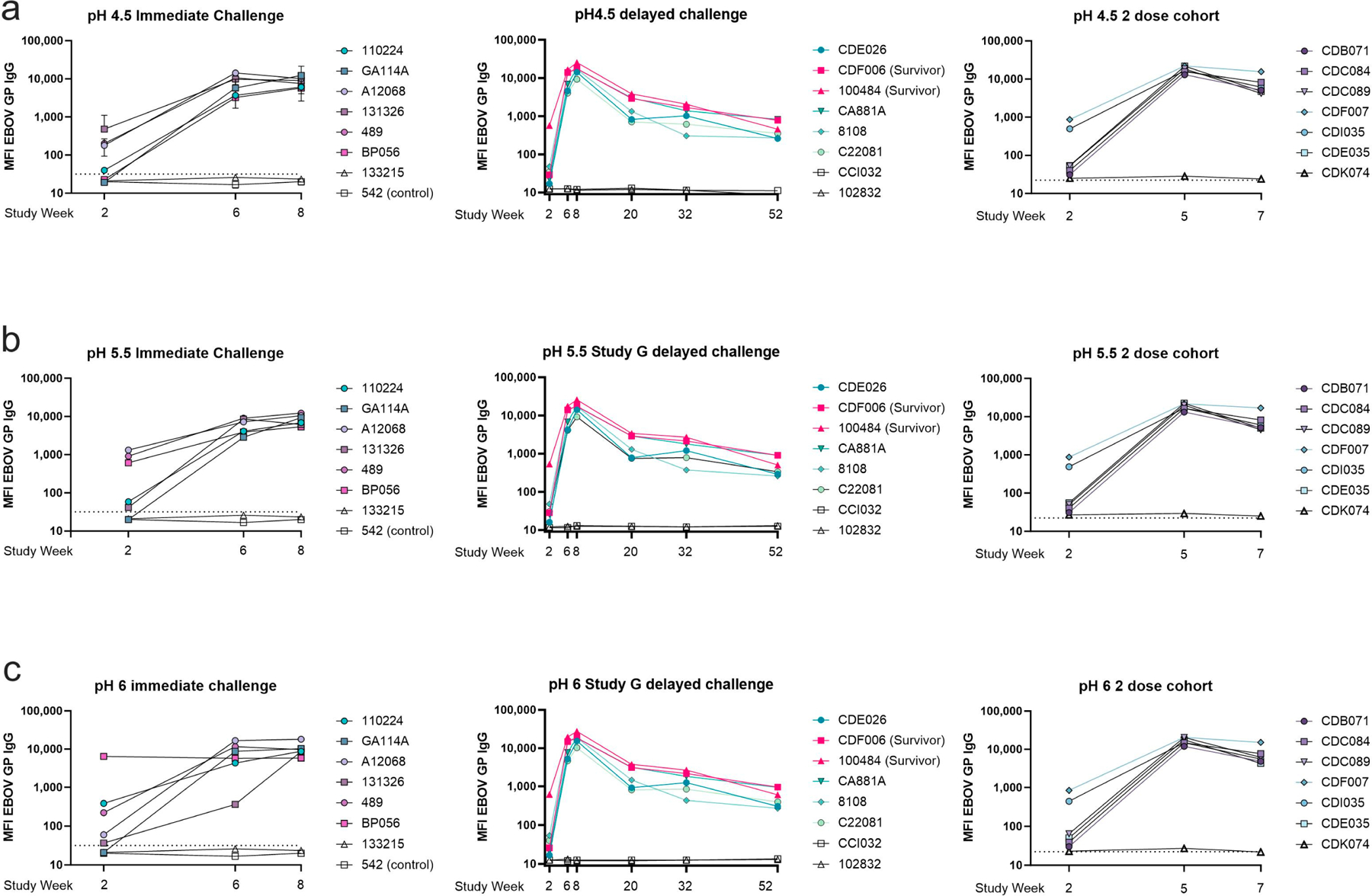
Antibody–antigen binding under acidic pH conditions. Antigen-specific IgG measured after incubation at various acidic pH. Serum was collected at various timepoints during the vaccination schedule and prior to viral challenge as indicated on the X-axis. Samples were diluted 1:10,000 and subjected to MIA with an acidic wash step with one of three buffers. (**a**) PBS buffered to pH 4.5; (**b**) PBS buffered to pH 5.5; (**c**) PBS buffered to pH 6. For animals challenged at week 10, week 62, and in the two-dose cohort respectively.

To assess binding strength at the pH experienced within an endosome (pH 4.5), we subjected the serum antigen-bound bead samples in the MIA to a wash in PBS buffered to either pH 6, 5.5, or 4.5. Wash with PBS buffered to pH of 7.4, the same as the assay buffer, was used as a negative control. The three-dose cohort demonstrated strong binding under all pH conditions (Figure 8). At pH 4.5 in the immediate challenge cohort, binding was highly variable after one dose; however, by the third dose, all animals reached similarly high antigen-specific antibody levels after the wash. The three-dose delayed challenge cohort developed similarly strong low pH antigen-binding antibody levels (Figure 8a). During the one-year rest period, antibody binding strength in most animals that did not survive the viral challenge decreased by week 20. Two survivors and one non-survivor exhibited strong binding at pH 4.5 at week 20 and 32, but this response waned by week 52, in which all animals showed a distinct decrease in overall binding. At week 52, there was no difference in binding between survivors and non-survivors. In the two-dose vaccine cohort, two out of the six vaccinated animals exhibited relatively strong low pH antigen–antibody binding after vaccine dose 1; the other four animals showed weak antigen–antibody binding just above the negative cutoff at this time. By the second dose, all animals had similar antibody binding strength at low pH. Prior to viral challenge, the two-dose cohort animals exhibited a decrease in antibody binding strength, but one animal, CDF007, had slightly increased binding at pH 4.5 compared to the other animals (Figure 8a). At pH 5.5 binding in the three dose, the immediate challenge cohort was again varied at week two; however, after the second and third doses, all animals developed strong antigen–antibody binding at pH 5.5. In the three-dose delayed challenge cohort, animals developed strong binding at pH 5.5; however, binding strength decreased at week 20. Animals that did not survive the viral challenge had lower pH-resistant IgG binding than the two surviving animals. This difference continued to week 32, but by week 52, all animals exhibited relatively similar binding strength. In the two-dose cohort, a similar trend in antibody binding strength was seen at pH 5.5 as at pH 4.5. Two animals in the multivalent cohort exhibited slightly stronger binding at week 2 (animals CDI035, CDF007); however, by the second dose, all animals developed strong antibody binding. Binding strength at week 7 was slightly lower than after the second dose (week 5) (Figure 8b). At a pH of 6, antibody binding after the first dose was again highly variable as it was at week two in both of the three-dose cohorts, but the same two animals in the two-dose cohort that exhibited stronger binding than the rest at pH 4.5 and 5.5 also exhibited stronger binding at pH 6. One non-survivor with high titers also demonstrated similar binding strength (Figure 8c).

### 3.7. Relative Avidity Index

**Figure 9.**
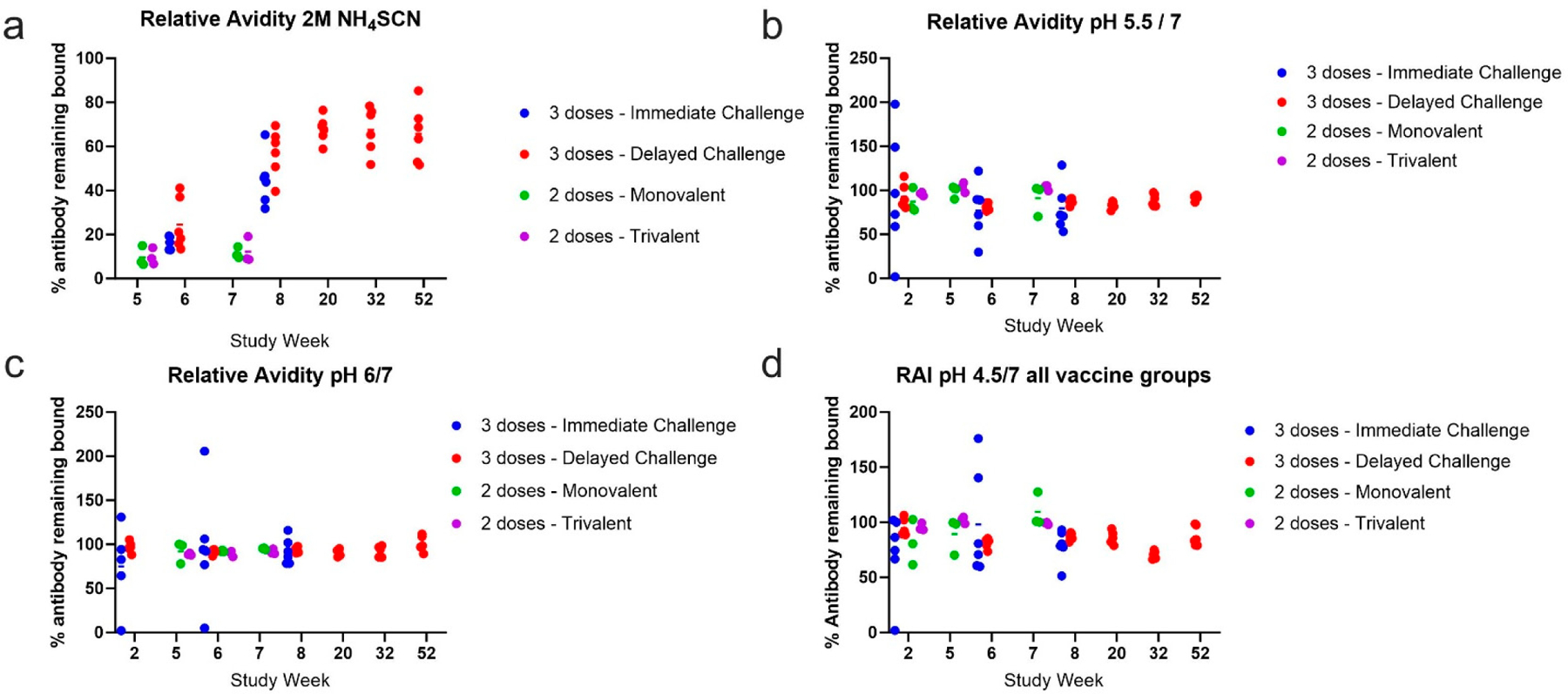
Relative avidity index. Relative avidity of each treatment type for each vaccine cohort. Relative avidity was calculated taking the ratio of the average MFI after treatment over the average MFI without treatment ×100%. RAI was plotted for each timepoint within the entire study. Each dot represents an animal within the respective vaccine cohort. (**a**) Relative avidity of 2 M NH4SCN treatment. (**b**) Relative avidity of pH 5.5 treatment. (**c**) Relative avidity of pH 6 treatment. (**d**) Relative avidity of pH 4.5.

To determine the relative binding strength (avidity), the ratio of bound antibody levels after exposure to a chaotrope or at the different pH conditions compared to antibody levels in untreated samples was calculated. We selected a concentration of chaotrope that was capable of decreasing antibody levels in all samples at peak antigen-specific antibody levels, 2 M NH4SCN, for this analysis. The percent of antibody remaining bound was plotted for each sample with levels above the negative cutoff. The relative avidity for 2 M NH4SCN after the second dose of vaccine in all groups was less than 40% (Figure 9a). In the threedose cohorts, about 30–70% of antibody remained bound after treatment two weeks after the third vaccine dose, but bound antibody levels increased to about 50–80% at study weeks 20–52. At low pH conditions, relative avidity was high for all timepoints and at pH 6, 5.5, and 4.5 (Figure 9b–d). While there was variability between animals, most animals showed high avidity at each pH condition tested. During the one-year rest period, the relative avidity index (RAI) increased after week 20, suggesting that as antigen specific titers waned, antibody that can bind strongly to EBOV GP remained, resulting in a higher RAI.

### 3.8. Avidity Does Not Correlate with Survival or Survival Day

**Figure 10.**
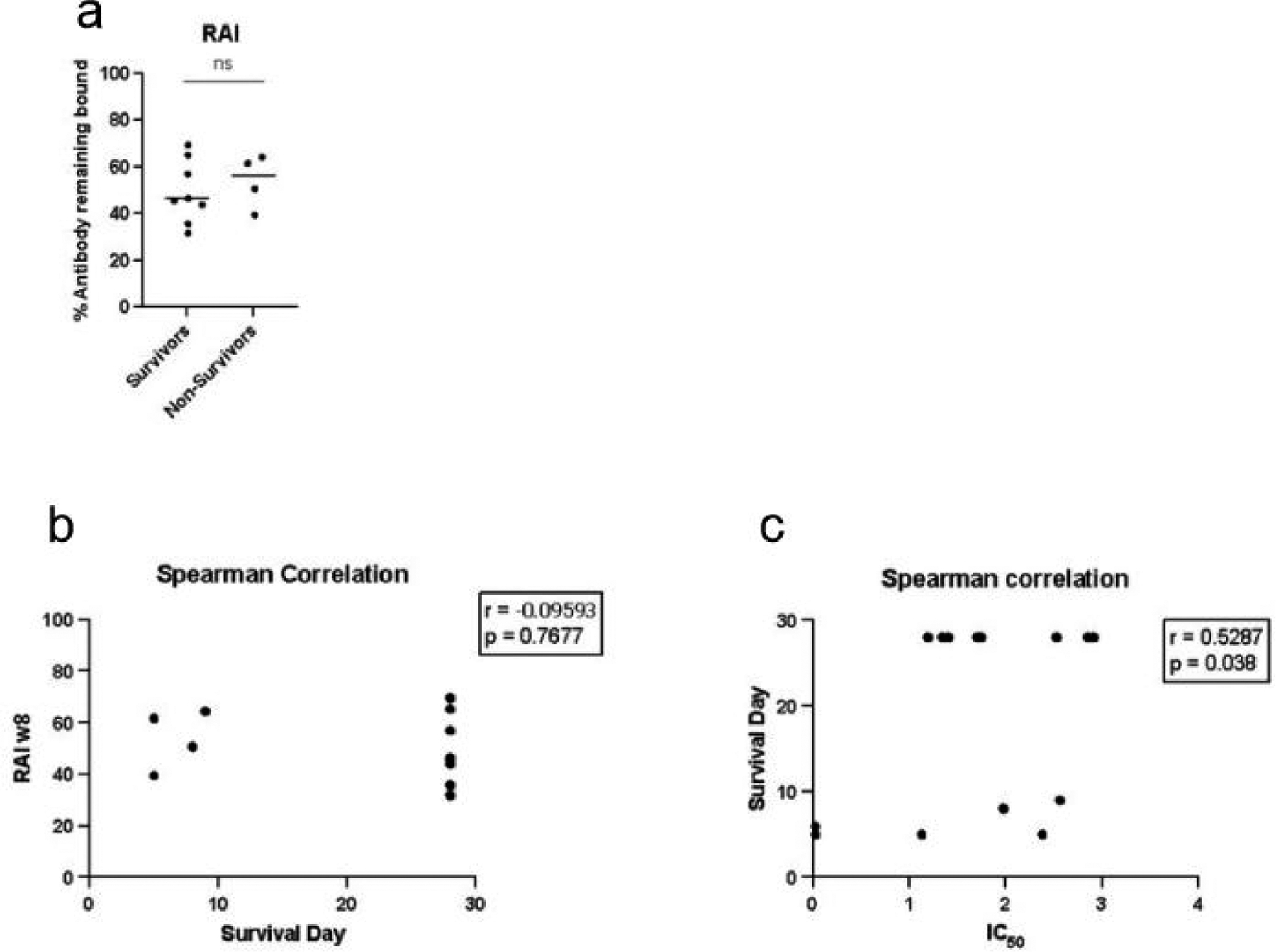
Avidity does not correlate with survival or survival day. (**a**) Statistical analysis (*t* test using the Mann–Whitney method) was conducted to compare relative avidity of anti-EBOV GP IgG detected at week 8 between survivors and non-survivors. (**b**) Spearman correlation of relative avidity calculated at study week 8 and survival day. (**c**) Spearman correlation of IC50 calculated at study week 8.

To determine whether antibody avidity plays a key role in protection, we conducted Mann–Whitney *t* tests and Spearman correlations between the relative avidity with 2 M NH4SCN and survival or survival day for each vaccine cohort (Figure 10). We conducted *t* tests comparing the relative avidity with 2 M NH4SCN at study week 8 between survivors and non-survivors, which did not yield statistically significant differences (Figure 10a). No correlation was found between the relative avidity and survival day (Figure 10b). The concentration of NH4SCN, which inhibited 50% of antibody binding (IC50), was calculated for animals in the three-dose cohort at study week 8. Spearman correlation of the IC50 and survival day yielded a slight correlation between these parameters, *r* = 0.5287; *p* = 0.038 (Figure 10c).

## 4. Discussion

Antibody binding strength is important for neutralizing the virus. Avidity varied with number of immunizations and time since last vaccination and, as expected, was enhanced with increased antibody maturation. Here, we demonstrated that multiple doses of vaccine enhance avidity maturation, as after three doses of vaccine, most animals maintained antigen-binding antibody concentrations well above the negative cutoff with treatments as high as 6 M NH4SCN. While a distinction between survivors and non-survivors could not be made based on post-dose 3 avidity, these responses were not surprising given the high rate of protective efficacy of this vaccine shortly after the third dose. These vaccines also elicit a cross-reactive response towards SUDV GP. However, after 20 weeks, these responses begin to increase in chaotrope sensitivity, indicating that the responses elicited by the three-dose regimen begin to wane (Appendix A). The cross-reactive response towards MARV GP exhibits notable waning; however, the SUDV GP cross-reactivity remains relatively stable over one year, showing a clear antigenic relationship between EBOV and SUDV, suggesting that the durability of this vaccine could be due to the immune response being directed towards conserved regions on the ebolavirus glycoproteins. Given that MARV GP and EBOV GP share 31% identity [15], the cross-reactivity of these polyclonal responses likely overlap within this range of identity between the two genera of filovirus with an increased overlap between species of the same genus (EBOV and SUDV). The antibodies that remain one year post challenge are of moderate avidity. In the three-dose delayed challenge cohort, the survivor animals and one non-survivor exhibited similar relative avidity (Figure 9). This result indicates that the overall binding strength is not a variable capable of differentiating survivors from non-survivors. This could be due to the epitopes themselves. For example, if antibody is bound strongly to a non-neutralizing epitope, this would not play a role in viral neutralization but would be readily detectable using our assay. Consequently, we would find strongly bound antibodies that do not correlate with survival. Therefore, protective efficacy may be the effect of a combination of strongly binding antibodies, some of which target neutralizing epitopes. A more nuanced assessment of high-avidity antibody in combination with epitope mapping may reveal these differences. In the two-dose cohort, affinity and avidity maturation did not occur to the same degree; this cohort had lower titers overall and exhibited a waning response by week 7. The three-dose vaccine cohorts developed stronger binding antibody after the third dose, suggesting that with continued affinity maturation, the composition of peripheral antibody contains an increased proportion of antibody with strong binding to the EBOV GP. While the two-dose cohort was not completely protected, avidity by an anionic chaotrope or by pH condition was not a measure that differentiated survivors from non-survivors. This is contradictory to the findings of Warfield et al. [16], where binding at low pH by antibody elicited from a VLP-based vaccine platform correlated with survival in non-human primates. In their analysis, binding at pH 4.5, 5, or 6 relative to binding at pH 7 was stronger in survivors than in non-survivors. While the VLP platform utilized a two-dose schedule, both our two-dose and three-dose schedules elicited antibody capable of strong binding at acidic pH. It is worth noting the variation in survival between these two studies: the VLP platform resulted exclusively in partially protected cohorts, while our vaccine platform generated complete protection of the cohort challenged 4 weeks post-dose 3.

In the lack of definitive correlates of protection lies a particular challenge in accurately measuring and predicting the protective efficacy of ebolavirus vaccines in humans. The strength of antibody binding posed a unique and potential avenue for determining protection against viral challenge. Our vaccines elicited strongly bound antibody in both survivors and non-survivors. Regarding the two-dose cohort, each animal experienced a low level of RNAemia, but only the non-survivor developed detectable viremia (unpublished data) and showed moderate avidity. The three surviving animals in this cohort developed moderate avidity, not much higher than animals that did not survive viral challenge and no correlations to survival day were found in this analysis.

A potential limitation of our study lies in the paratope diversity elicited by using a near full-length glycoprotein. The use of an anionic chaotrope directly interacts with the paratope–epitope bond, which can result in variable chaotrope sensitivity based on the level of structure of the epitope. Other experiments from our studies (data not shown here) indicated that neutralizing antibody correlated with survival day in the delayed challenge cohort; however, the thiocyanate treatment demonstrated that survivors had moderate avidity at best. This could be due to increased chaotrope sensitivity of neutralizing epitopes. Complementary pH-based assays determined that two doses of vaccine elicited antibody capable of remaining bound to GP at a pH representative of an endosome (pH < 6). This finding is key to understanding the potential for mitigating host–viral membrane fusion. These findings indicate that a protein subunit vaccine is capable of eliciting a protective humoral response and one that can potentially neutralize in a variety of physiological spaces such as mucosal tissue (pH 6) and endosomes (pH 5.5, 4.5). Considering the pH sensitivity of EBOV GP, antibody remaining bound in these various pH conditions can potentially prevent the GP fusion domain from being able to fuse with the endosomal membrane, as demonstrated by the mechanism of protective efficacy of monoclonal antibody immunotherapies. However, as indicated by the extensive studies of ZMapp, the binding epitope may also be quite relevant in an endosome, considering the newly exposed epitopes resulting from cathepsin cleavage. These findings indicate the need for future epitope studies of the humoral responses elicited using filovirus vaccines to further understand antibody–antigen complexes under these pH conditions.

Challenges in correlating relative avidity to survival day could be due in part to the small number of animals (“n”) of each study, as is typical for nonhuman primate studies. We were able to demonstrate a weak correlation between the NH4SCN IC50 at study week 8 and survival day; this is a relationship for further investigation. An expanded investigation of more vaccine cohorts with variation in protective efficacy could enhance the analysis reported here. We conducted an analysis of pre-challenge sera one year after immunization; however, a measure of a recall response, such as a post-challenge antibody characterization or a post-booster dose response may provide more information regarding its relevance to protective efficacy. Our vaccines generated high antibody concentrations with strong binding at low pH and moderate avidity as measured by NH4SCN treatment. Future studies should investigate the avidity to specific neutralizing epitopes to determine the proportion of high-avidity, low-pH binding, neutralizing antibody to further elucidate the protective efficacy of the humoral response. A study on FcR binding and recruitment of effector cells would also be helpful in determining the role of complementary non-neutralizing antibody functions.

## Figures and Tables

**Table 1.** Vaccine cohorts used in this study.

Formulation	Doses	Challenge	*n*	Survivors	Non-Survivors
25 μg EBOV GP + CoVaccine HT^™^	3	EBOV—week 10 challenge	6	6	0
25 μg EBOV GP + CoVaccine HT^™^	3	EBOV—week 62 challenge	6	2	4
25 μg EBOV GP + CoVaccine HT^™^	2	EBOV	4	3	1
25 μg EBOV GP + 25 μg MARV GP + 25 μg SUDV GP + CoVaccine HT^™^	2	EBOV	3	0	3

**Figure A1.**
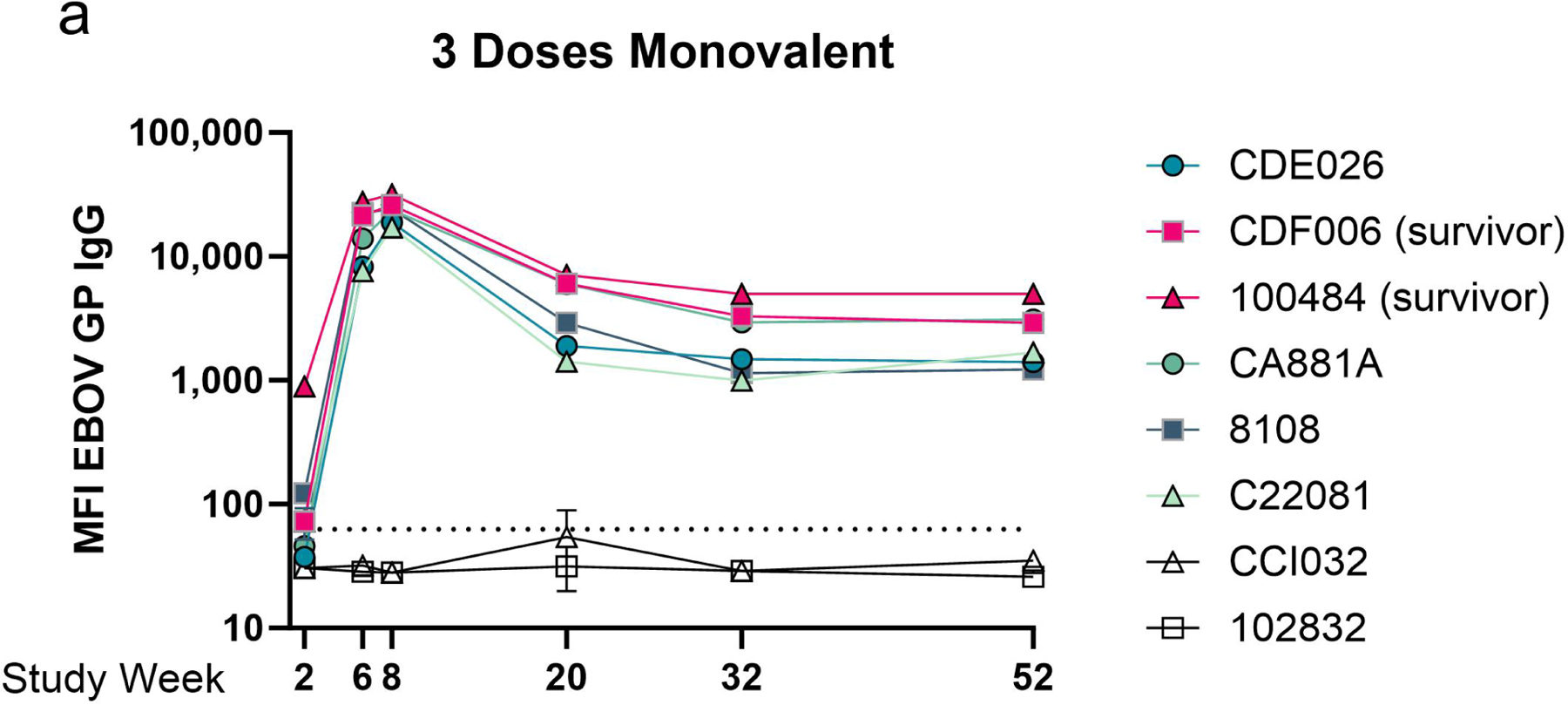

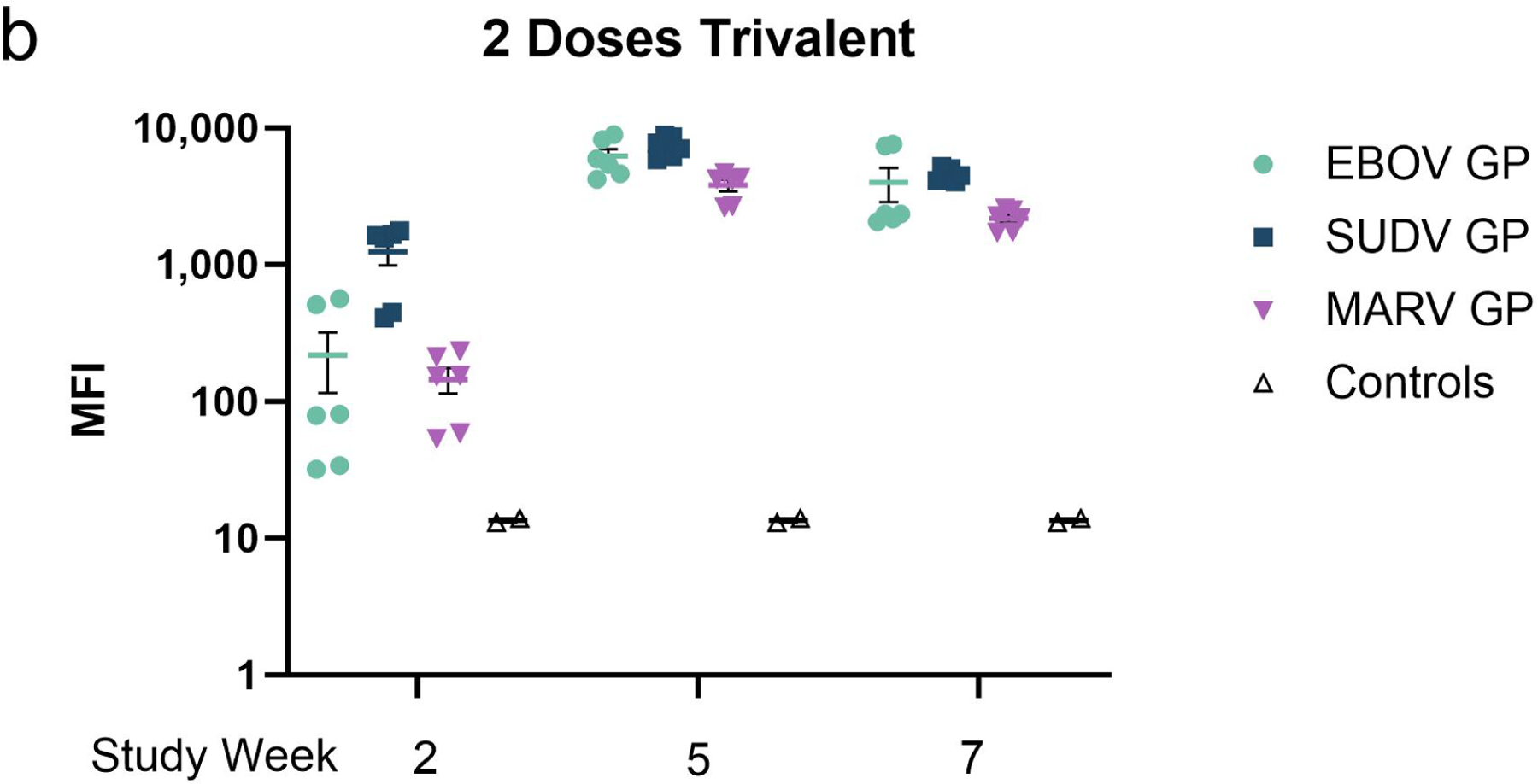
Untreated antigen-specific IgG levels. (**a**,**b**) Serum samples collected were diluted 1:10,000 and subjected to MIA assay. Datapoints represent the mean of replicate assays with error bars of the standard deviation of individual animals. (**a**) MFI of EBOV GP-specific IgG. In a three-dose cohort, serum samples were collected two weeks after each dose and intermittently for one year. Eight animals were challenged at week 62, including two controls (nonvaccinated). (**b**) MFI of antigen-specific IgG in a two-dose trivalent cohort. Six animals were given vaccine and two served as unvaccinated controls. Serum samples were collected two weeks after each dose of vaccine. Datapoints represent the mean MFI of replicate assays per animal for each antigen included in the vaccine. The line represents the mean of all datapoints with error bars representing the standard deviation. All samples were run in duplicate.

## Data Availability

The raw data supporting the conclusions of this article will be made available by the authors without undue reservation.
